# The Social Side of Internet of Things: Introducing Trust-Augmented Social Strengths for IoT Service Composition

**DOI:** 10.3390/s25154794

**Published:** 2025-08-04

**Authors:** Jooik Jung, Ihnsik Weon

**Affiliations:** 1 Airport Industrial Technology Research Institute, Incheon International Airport Corp, Incheon Jung-gu Airport Rd. 424-47, Incheon 22382, Republic of Korea; jj87@airport.kr; 2 The Flexible Manufacturing R&D Department, Korea Institute of Industrial Technology, Incheon 21999, Republic of Korea

**Keywords:** Internet of Things (IoT), service composition, social internet of things (SIoT), social strength, trust, remote sensing

## Abstract

The integration of Internet of Things (IoT) systems with social networking concepts has opened new business and social opportunities, particularly by allowing smart objects to autonomously establish social relationships with each other and exchange information. However, these relations must be properly quantified and integrated with trust in order to proliferate the provisioning of IoT composite services. Therefore, this proposed work focuses on quantitatively computing social strength and trust among smart objects in IoT for the purpose of aiding efficient service composition with reasonable accuracy. In particular, we propose a trust-augmented social strength (TASS) management protocol that can cope with the heterogeneity of IoT and demonstrate high scalability and resiliency against various malicious attacks. Afterward, we show how the TASS measurements can be applied to service planning in IoT service composition. Based on the experimental results, we conclude that the proposed protocol is, in fact, capable of exhibiting the above-mentioned characteristics in real-world settings.

## 1. Introduction

The widespread adoption of smart objects and ubiquitous wireless connectivity has propelled the Internet of Things (IoT) to prominence, creating immense business and social opportunities. This trend heralds a new era in which billions of devices communicate and exchange information to meet human needs and perform myriad tasks [1,2]. Moreover, researchers envision that future IoT systems will incorporate social networking principles, allowing smart objects to interact in human-like social patterns—a concept known as the Social Internet of Things (SIoT) [1,3,4,5].

In essence, smart objects in an SIoT can autonomously establish peer-to-peer relationships with other objects, effectively forming device social communities [6]. Within these social circles, the objects can play the role of either a service provider or a service requester or both. It is anticipated that many SIoT applications will be built on service-oriented architectures [1,7]. However, realizing this vision requires significant effort to transition from traditional Web service environments to the SIoT context. Established methods for service discovery, composition, and recommendation must be rethought to accommodate the distinctive features of SIoT networks [8]. As a result, a pressing challenge is to develop a trustworthy SIoT ecosystem that can reliably support these autonomous interactions [9,10,11,12].

The motivation of providing a trust management protocol for an SIoT system is quite clear, as shown below:

- ***Trust-Related Attacks:*** Malicious or misbehaving objects might execute various trust-related attacks, threatening to undermine the entire service provisioning process in an SIoT system [13].
- ***Dishonest Social Relationships:*** Malicious nodes could also exploit their close social ties with honest objects, forming deceptive or dishonest “friendship” relations within the network [14].

Despite the clear need for improved trust management, developing a protocol for SIoT faces several key obstacles:

- ***Scalability and Resource Constraints:*** SIoT environments typically involve a vast number of heterogeneous smart objects, many of which have limited storage and computational resources. Existing trust management schemes often struggle to scale under these conditions, as they were not designed for networks of this size and device diversity.
- ***Dynamic Topology:*** Smart objects in SIoT are mobile; nodes may frequently join or leave the network [15]. A robust protocol must accommodate this dynamism by enabling an efficient exchange of trust information and allowing newly joined objects to rapidly establish trust relationships—all while maintaining a reasonable level of accuracy in trust estimation.
- ***Malicious Behavior and Resilience:*** Adversarial nodes might attempt to sabotage others’ reputations or fraudulently boost their own. Countering such behavior demands a trust management solution that is sustainable and resilient against various malicious attacks. In particular, the system should defend against reputation manipulation and ensure trust scores remain reliable [16,17].

In light of the above challenges, our work introduces a new trust management framework for SIoT. The goal is to effectively compute trust values among IoT objects and to leverage these values for better service composition. We also propose a method to quantitatively evaluate social strength between objects, which we later combine with the trust values. By integrating trust with social relationship metrics, TASS is designed to cope with the heterogeneity of IoT devices while demonstrating high scalability and resilience against malicious behaviors. In what follows, we outline the two main components of TASS—social strength computation and trust computation—and how they are unified for service composition.

The first component of TASS calculates the social strength of inter-object relationships using an information-theoretic approach. Specifically, we leverage entropy-based metrics to capture temporal usage patterns and incorporate the diversity of co-usage events in our analysis. In our design, we use Rényi entropy to quantify the affinity between two objects based on their shared usage history, and we extend this with Rényi diversity to account for variability in these co-usage interactions. We further address the issue of sparse data by incorporating spatial context: considering the geographical distance between object locations as a factor. In particular, we employ the Mutually Nearest Distance (MND) metric, which effectively captures the spatial distribution overlap of two sets of locations. Two points (object locations) are defined to be mutually nearest if each is the closest neighbor of the other—that is, each object is the nearest neighbor of the other within their respective location sets. Using this criterion, we iteratively match pairs of objects and compute the MND, allowing every point to participate in the matching and thereby producing a one-to-one pairing between the location sets [18]. By combining temporal, diversity, and spatial insights, our social strength measure provides a robust quantitative indicator of how “closely related” or co-workable any two smart objects are.

The second part of the proposed TASS deals with estimating and managing trust values among objects. Here, we formulate two types of trust, namely direct trust and indirect trust. Direct trust is a measure of object satisfaction experience after interacting with a particular service provided by an object. On the other hand, indirect trust represents a recommendation score from other objects when the service requestor has no past interaction with the service provider. Finally, the proposed TASS integrates the social strength value with the trust to reach the ultimate TASS score, which will be used in IoT service composition. Our main contributions are summarized as follows:

- We formally define the terms social strength and trust in the context of SIoT, with their application in service composition.
- We develop a trust-augmented social strength computation algorithm which quantitatively measures the strengths of inter-object social relationships based on trust scores.
- The proposed TASS algorithm is scalable to large SIoT systems and can effectively handle the heterogeneity issue in SIoT.
- Our experimental results based on a real-world dataset show that the TASS algorithm can support accurate and efficient SIoT service composition, and provide resiliency against trust-related attacks.

The remaining sections are organized as follows. In Section 2, we present a brief survey of related work. Section 3 discusses various possible malicious behaviors of a node in SIoT. Section 4 introduces the proposed trust-augmented social strength and how it is computed. After that, Section 5 describes how TASS can be exploited in IoT service composition. Then, Section 6 discusses the results and a thorough analysis of our experimentation in detail. Finally, we conclude this paper and discuss our plans for future work in Section 7.

## 2. Related Work

Recently, the Social Internet of Things (SIoT) and its associated trust management protocols have garnered significant research interest. One closely related study is presented in [19,20,21], where the authors introduce an adaptive trust management protocol for SIoT systems that defends against various types of malicious behavior. The protocol incorporates multiple trust-related dimensions—honesty, cooperativeness, community interest, and centrality—to compute trust scores. These scores are dynamically updated through an adaptive control mechanism, enabling the system to maintain resiliency under attack scenarios.

Another notable contribution is found in [22], which identifies key trust attributes that reflect the honesty of smart objects. Based on these attributes, the authors propose a subjective computational model that leverages experience and opinions from friendly objects. The final honesty score is then computed using a machine learning algorithm. However, a significant limitation of this approach is its lack of robustness against adversarial behavior within SIoT environments.

In refs. [23,24], the authors propose TRM-SIoT, a trust model that mathematically encodes the prior interaction history of smart objects into representative trust scores. This model combines centralized and distributed reputation mechanisms to form a hybrid trust system. While it adapts concepts from peer-to-peer (P2P) and mobile ad hoc networks to the SIoT domain, it does not adequately address scalability—an essential requirement for large-scale SIoT deployments.

The EigenTrust algorithm [25], originally developed for P2P systems, is another foundational trust mechanism. It computes a global trust score for each node by aggregating trust recommendations, weighted by the trustor’s confidence in the recommenders. This system assumes the presence of trustworthy nodes immune to malicious influence, thereby facilitating rapid trust convergence and protection against deceitful behavior.

Similarly, PeerTrust [26] provides a reputation-based framework for evaluating peer trustworthiness in P2P environments. The model integrates recommendation feedback with peer transaction history and considers factors such as transaction context, recommender credibility, and social proximity. However, to be applicable in SIoT, this framework would require substantial modification to account for device heterogeneity and dynamic topology.

In [27], the need for trust management in information-centric networking (ICN) is explored. The authors propose the Fast and Efficient Trust Management Scheme (FETMS), which evaluates trust based on both communication and data reputations. Communication trust is computed through direct and indirect observations within the network, while data trust is assessed based on transmission integrity.

A similar hybrid trust approach is found in [28], where direct and indirect trust are combined for vehicular IoT environments. In this case, fuzzy logic is used to assess direct trust, and reinforcement learning techniques are employed to infer indirect trust for unobserved vehicles. Although the method achieves high precision and recall in detecting malicious actors, it faces challenges related to data synchronization across distributed systems and the fast-changing dynamics typical of vehicular networks.

Finally, recent efforts have focused on integrating blockchain technology with IoT trust infrastructures. For example, in [29], the authors propose IoT Passport, a blockchain-based cross-platform collaboration framework. The system comprises both local and global trust domains, where local domains manage intra-organizational resources and global domains facilitate inter-organizational collaboration. Trust among entities is established through smart contracts governed by cross-platform access control policies [30].

Previous studies have shown limitations in terms of trust estimation accuracy and adaptability to various network conditions. In particular, real-time handling of device heterogeneity and dynamic network topology has been insufficient. This study addresses these gaps and highlights the necessity of the proposed trust-augmented social strength (TASS) protocol.

## 3. Malicious Behaviors in SIoT

Before introducing our method for computing trust-augmented social strength, it is essential to identify several malicious behaviors that can compromise the overall trustworthiness of Social Internet of Things (SIoT) systems. In general, malicious nodes exhibit socially uncooperative behavior that undermines the foundational integrity of SIoT networks [29,31]. The following are some of the most prominent types of trust-related attacks observed in SIoT environments:

***Self-promoting Behavior:*** In this scenario, a malicious node attempts to artificially boost its own reputation by issuing positive recommendations about itself. The goal is to increase its chances of being selected as a service provider. However, once selected, the node often delivers degraded or faulty service. For example, a malicious smart vehicle might broadcast misleading route information to other nearby vehicles to manipulate traffic conditions in its favor. Such actions can disrupt traffic flow and pose serious consequences for critical services like emergency vehicle navigation.

***Bad-mouthing Attacks:*** This type of attack involves a node deliberately damaging the reputation of well-behaved nodes by issuing unfairly negative recommendations. The intent is to reduce the likelihood that legitimate nodes will be selected for service roles. Often, multiple malicious nodes collaborate to intensify the impact of these false evaluations, making it more difficult to distinguish between honest and dishonest actors.

***Ballot-stuffing Attacks:*** Contrary to bad-mouthing, ballot-stuffing involves inflating the reputation of a malicious node by providing an excessive number of positive recommendations. This tactic is usually carried out through collusion among a group of adversarial nodes. The collective manipulation significantly increases the targeted node’s chance of being selected, despite its untrustworthy behavior.

***Oscillating Behavior:*** In oscillating attacks, a malicious node intentionally alternates between cooperative and uncooperative behavior across different services or interactions. For instance, it may act honestly in one context while behaving maliciously in another. While such behavior may be easier to detect when it occurs across different services, identifying it within the same service is considerably more complex. This inconsistency creates conflicting observations among honest nodes, leading to confusion and inaccuracies in collective reputation assessments. Ultimately, even well-behaved nodes may suffer from reduced trust scores due to the disruptive influence of oscillating attackers.

## 4. Trust-Augmented Social Strength

### 4.1. Social Strength in SIoT

The first component of the TASS framework focuses on computing social strength values by combining entropy and spatial distance metrics. In this study, we utilize human–object interaction data as the primary input for this computation. It is important to clarify that our research does not aim to design the system architecture responsible for collecting, managing, or updating these data streams. Rather, our primary objective is to develop a quantitative metric that effectively captures the degree of “closeness” or relational proximity between two smart objects. In this context, human–object interaction is defined as a sequence of discrete events where an individual engages with a particular smart device, formally described as follows.

**Definition** **1 (Object Usage Vector).** *An object usage vector captures the historical interaction patterns of a smart object with users over time. It contains the user IDs and a list of timestamps at which the object is accessed. The general format of the object usage vector of Object i is Vi = (<t1,1, …, t1,i1>, …, <tN,1, tN,2, …, tN,iN>), where N denotes the number of users.*

Once we have the object usage vectors properly assigned to each smart object, we formulate another type of vector as follows.

**Definition** **2 (Co-usage Vector).** *The combined interaction history of two smart objects is represented by a co-usage vector. Specifically, when both objects are accessed by the same user within a predefined time interval, they are considered to be co-used. The general form of the co-usage vector for Objects i and j is given by*

*Wij = (wij,1, wij,2, …, wij,N),*

*where wij,u—referred to as the local frequency—denotes the number of co-usage events between Objects i and j by User u.*

Accordingly, the main objective of this subsection is to estimate the social strength between each pair of smart objects based on their corresponding co-usage vector. Although this concept has been referenced, a formal definition of social strength in the context of SIoT has not yet been provided. We define social strength as a quantitative measure that reflects the ability of two smart objects to collaborate effectively in performing tasks for users. This is also referred to as their co-workability.

In order to compute the social strength between two smart objects, we first utilize the Renyi entropy and diversity. These two measures are adopted from the work in [32,33] and revised so that it can be applied in the context of SIoT environment.

Let $c_{i,j}^{u,t}=\langle i,j,u,t\rangle$ be a co-usage of Objects *i* and *j* by User *u* at time *t*. In addition, $C_{ij}^{u}=\cup_{t}c_{i,j}^{u,t}$ is the set of co-usages of Objects *i* and *j* by User *u*. With a little tweaking to the equation, $C_{ij}=\cup_{u,t}c_{i,j}^{u,t}$ then becomes the set of all co-usages of Objects *i* and *j* by all users. Based on these definitions, the probability that a randomly picked co-usage from the set $C_{ij}$ belongs to User *u* is as follows:

(1) $$P_{ij}^{u}=\frac{\left|c_{ij}^{u}\right|}{\left|c_{ij}\right|}$$

Suppose we randomly select a co-usage event from the set $C_{IJ}$, which contains all instances where Objects i and j were accessed together by users. If we then define the user associated with this randomly selected event as a discrete random variable, the level of uncertainty regarding which user performed the co-usage can be quantitatively captured using Rényi entropy. This entropy, computed for the co-usage interactions between Objects i and j, reflects the diversity of user participation in their joint usage patterns. A higher entropy value indicates a more uniform distribution of co-usages across users, while a lower value suggests concentration among fewer users. The formal definition of Rényi entropy for Objects i and j is given in Equation (2).

(2) $$H_{ij}^{R}=\frac{1}{1-\alpha }\log\left(\sum_{u}{\left(P_{ij}^{u}\right)}^{\alpha }\right)=\frac{1}{1-\alpha }\log\left(\sum_{u}{\left(\frac{w_{ij,u}}{f_{ij}}\right)}^{\alpha }\right)$$

where the upper index *R* denotes Renyi, and $f_{ij}=\sum_{u}w_{ij,u}$ represents the *global frequency* of Objects *i* and *j*, which is basically the sum of all co-usages of Objects *i* and *j*. Moreover, *α* ≥ 0 determines the entropy’s sensitivity to the local frequency values, hence controlling the impact of extreme co-usages.

The next step is to integrate the Renyi entropy with the concept of diversity, eventually producing Renyi diversity. This new measure for quantifying social strength is depicted in (3).

(3) $$\begin{array}{l} D_{ij}^{R}=\exp\left(H_{ij}^{R}\right)=\exp\left[\frac{1}{1-\alpha }\log\left({\sum_{u}\left(P_{ij}^{u}\right)}^{\alpha }\right)\right]\\ ={\left(\sum_{u}{\left(P_{ij}^{u}\right)}^{\alpha }\right)}^{1/\left(1-\alpha \right)}={\left(\sum_{u}{\left(\frac{w_{ij,u}}{f_{ij}}\right)}^{\alpha }\right)}^{1/\left(1-\alpha \right)} \end{array}$$

Thus far, we have introduced a quantitative metric for estimating the social strength between pairs of smart objects, which serves as the foundation for subsequent analysis and can be further refined as the framework evolves. This metric fundamentally relies on two key aspects of object interactions: temporal dynamics and usage diversity.

The temporal component is embedded in the construction of co-usage vectors, which are derived from raw object usage logs. Specifically, a co-usage event is recorded whenever two distinct objects are accessed by the same user within a predefined time window. This temporal proximity reflects a likely functional relationship between the objects, as it suggests that the user is utilizing them in a coordinated manner to accomplish a particular task.

On the other hand, diversity is captured by analyzing the distribution of these co-usage events across the entire user population. Using entropy-based formulations—particularly Rényi entropy and its derived diversity measure—we quantify how broadly the co-usage behavior is shared among different users. A higher diversity score indicates that the observed co-usage is not limited to a few individuals, but is instead a common behavior pattern among many users, implying general co-functionality and usefulness of the object pair. These two factors—temporal alignment and user diversity—are grounded in the following behavioral intuitions:

***Temporal Feature Intuition:*** When users perform tasks involving multiple objects, they are likely to access those objects within a short time span. The closer the access times, the stronger the likelihood that the objects are functionally related.

***Diversity Intuition:*** If two objects are frequently co-used by a wide range of users, this implies their combined functionality is generally beneficial, and not just task-specific for a few individuals.

However, our current formulation does not yet incorporate spatial characteristics of object usage—a factor that can significantly influence interaction patterns in real-world IoT environments. Geographical context plays a crucial role in human–object interactions, especially in settings such as smart homes or public facilities.

***Spatial Feature Intuition:*** Users are naturally more inclined to interact with objects that are physically proximate. The closer two objects are in physical space, the more convenient and likely it is for a user to utilize them together during a single session of activity. Ignoring spatial proximity may lead to underestimation of certain co-usage patterns or overestimation of others that are less feasible in practice due to location constraints.

Incorporating spatial proximity into the social strength metric allows for a more comprehensive understanding of how physical arrangement influences perceived and practical object relationships.

Moreover, many modern IoT devices are inherently mobile, frequently changing their physical locations as users move through different environments. This mobility further emphasizes the importance of incorporating spatial information into the social strength computation, as the relative proximity of objects may vary over time and influence interaction patterns dynamically.

Additionally, one of the practical challenges in real-world deployments is the limited availability of comprehensive object usage datasets. In many cases, the data collected are sparse, with relatively few co-usage instances recorded for any given pair of objects. This data sparsity can result in low Rényi diversity values, which in turn may reduce the discriminative power of the proposed social strength metric. When diversity values are disproportionately low due to insufficient observations, the reliability of co-usage-based inferences is compromised, potentially leading to underestimation of valid social ties among objects.

To compensate for the issues, we introduce another measure for social strength. We adopt the Mutually Nearest Distance (MND) [18] and integrate it with the Renyi diversity.

Given two sets *X* and *Y*, both containing the same number of points, a *matching* between *X* and *Y* is a subset μ⊆X×Y of pairs of points (one from each set). To rephrase this, if $\left(x,y\right)\in \mu$, where *x* and *y* are points belonging to the sets *X* and *Y,* respectively, then we say that *x* is *matched* with *y* and vice versa. Now, we define what it means for two points to be *mutually nearest*. Using the same notations, we state that *x* and *y* are *mutually nearest* if *x* is the nearest neighbor of *y* out of all points in *X,* and *y* is the nearest neighbor of *x* out of all points in *Y*.

Building on the two previously defined sets, we construct a mutually nearest matching by iteratively pairing points that are the closest to each other across the two sets. In each iteration, the algorithm identifies the pair of points—one from each set—that are mutually nearest, meaning each is the closest point to the other. Once a pair is matched, both points are removed from further consideration. This process continues until all points have been matched, resulting in a one-to-one correspondence between the two sets.

Although techniques exist for handling cases where the two sets differ in size, such scenarios are not applicable in our context. This is because each co-usage event between two objects yields exactly one location from each object, ensuring that the number of points in both sets is always equal.

Once we have a complete list of mutually nearest matchings, we compute the MND between the two sets of points, denoted by *d_MN_*(*X*,*Y*), using (4).

(4) dMN(X,Y)=∑(x,y)∈μMNdist(x,y)μMN

Here, $\mu_{MN}$ denotes the set of mutually nearest matchings between two location sets. Unlike many state-of-the-art approaches that rely on partial or selective matching, the Mutually Nearest Distance (MND) metric ensures that every point in both sets participates in the matching process, thereby contributing proportionally to the overall distance calculation. Specifically, if a set contains m points, each point is assigned a uniform weight of 1/m, ensuring equal influence on the final MND value.

We then incorporate the MND value into the local frequency of co-usages, resulting in an MND-weighted frequency metric. This augmented value captures not only how frequently two objects are co-used, but also the spatial proximity of their respective interaction locations, as defined below.

(5) $$f_{ij}^{MN}=\sum_{u}w_{ij,u}\times \exp(-norm(d_{MN}(l_{i,u},l_{j,u})))$$

where $f_{ij}^{MN}$ defines the MND-based frequency of Object *i* and Object *j*, and $w_{ij,u}$ denotes the local frequency of Objects *i* and *j* for User *u*. Also, $norm(d_{MN}(l_{i,u},l_{j,u}))$ denotes the normalized Mutually Nearest Distance value between the accessed locations of Object *i* and Object *j* for User *u*.

The two measures are then combined together to produce a final social strength value as shown below.

(6) $${ss}_{ij}=\beta \cdot D_{ij}^{R}+(1-\beta )\cdot f_{ij}^{MN}$$

where *ss_ij_* denotes the social strength between Objects *i* and *j*, and *β* corresponds to a weight value. Also, $D_{ij}^{R}$ and $f_{ij}^{MN}$ represent the Renyi diversity and MND-based frequency, respectively.

### 4.2. Trust in SIoT

The second part of TASS exploits direct trust and indirect trust to yield a representative trust score among objects. Almost all of the traditional approaches that utilize these two elements are targeted for humans such as service requestors who interact with various services. Consequently, we adopt and extend the methods in [34] so that they can be applied to the context of SIoT, where smart objects are the main protagonists.

To initiate the trust computation process, we first evaluate direct trust using a Bayesian framework [35]. Given its strong theoretical foundation and widespread adoption in trust management research, the Bayesian approach serves as a robust and reliable method for modeling direct trust in our system.

In SIoT, direct trust is primarily based on an object’s satisfaction experience after interacting with another object. So, we define *s_i_*_,*j*_ to be the current satisfaction experience of Object *i* toward Object *j*. We only consider a simple case in which the object satisfaction experience is a binary value, with 1 being satisfied (i.e., successful completion of a task) and 0 indicating not satisfied (i.e., unsuccessful task completion). In this particular case, we can consider *s_i_*_,*j*_ as an outcome of a Bernoulli trial with the probability of success parameter *θ_i,j_* following a Beta distribution. Here, the distribution is also known as a conjugate prior for the Bernoulli distribution, which can be denoted as Beta(*a_i_*_,*j*_, *b_i_*_,*j*_). Furthermore, the posterior *p(θ_i,j_*|*s_i_*_,*j*_) has a Beta distribution as well. Therefore, the hyper parameters *a_i_*_,*j*_ and *b_i_*_,*j*_ are updated based on trust decay and satisfaction experience as shown in (7).

(7) $$\begin{array}{l} a_{i,j}=e^{-\delta \Delta t}\cdot a_{i,j}^{\left(old\right)}+s_{i,j},\\ b_{i,j}=e^{-\delta \Delta t}\cdot b_{i,j}^{\left(old\right)}+1-s_{i,j} \end{array}$$

In the above equation, we utilize exponential decay, denoted as $e^{-\delta \Delta t}$, on $a_{i,j}^{\left(old\right)}$ and $b_{i,j}^{\left(old\right)}$ to model trust decay, where *δ =* 003 is a decay factor and Δ*t* represents the trust update interval. Usually, the decay factor is given a small number to produce small trust decay over time. It is also worth mentioning that *s_i_*_,*j*_ in the upper part of (7) contributes to positive observations, whereas 1 − *s_i_*_,*j*_ contributes to negative observations. Finally, the direct trust of Object *i* toward Object *j*, which is denoted by $T_{i,j}^{direct}$, can be computed as the expected value of *θ_i,j_* as illustrated below.

(8) $$T_{i,j}^{direct}=E\left[\theta_{i,j}\right]=\frac{a_{i,j}}{a_{i,j}+b_{i,j}}$$

In many studies, the values of *a_i_*_,*j*_ and *b_i_*_,*j*_ are often set to 1 when there is no prior knowledge available to begin with. In this work, however, we compare the social relationships of the two objects structurally by using Jaccard index, which is extremely popular in studies related to link prediction. Consequently, the initial values for *a_i_*_,*j*_ and *b_i_*_,*j*_ are set to *sim_Jaccard_*(*i*,*j*) and 1 − 1 − *sim_Jaccard_*(*i*,*j*), respectively. The equation below demonstrates how Jaccard index can be computed.

(9) $${sim}_{Jaccard}(i,j)=\frac{\left|\Gamma (i)\cap \Gamma (j)\right|}{\left|\Gamma (i)\cup \Gamma (j)\right|}$$

To reach the final trust score between two objects, we calculate the indirect trust between them. More specifically, the indirect trust is computed based on the recommendations from a third-party object. With a minor revision to (9), we measure the friendship similarity between two objects, namely a trustor object and a recommender object. Here, we simply replace the trustee Object *j* with a recommender Object *r*. Each object can periodically send a request and share trust recommendations with its neighboring (or “friend”) objects, or does so prior to initiating a service request. Upon receiving such recommendations, Object *i* identifies the top-*k* objects with the highest similarity scores relative to itself. It then calculates the indirect trust toward Object j based on the aggregated feedback from these k most-similar peers, as defined below.

(10) $$T_{i,j}^{indirect}=\sum_{r\in k}\frac{{sim}_{Friendship}(i,r)}{\sum_{r\in k}{sim}_{Friendship}(i,r)}\cdot T_{r,j}^{direct}$$

It is important to note that each trust recommendation is assigned a weight proportional to the similarity between the recommender and the requesting object. Specifically, the weight is calculated as the ratio of the recommender’s friendship similarity score to the total sum of similarity scores across all recommenders. This ensures that recommendations from more closely related (i.e., more socially similar) objects exert a greater influence on the indirect trust calculation.

After computing both the direct trust and indirect trust values, we integrate them to derive the final trust score from Object *i* to Object *j*. This overall trust score, denoted as *T_i_*_,*j*_, captures both Object *i*’s personal experience with Object *j* and the opinions of socially similar peers. The final trust is computed using the following equation:

(11) $$T_{i,j}=\gamma \cdot T_{i,j}^{direct}+(1-\gamma )\cdot T_{i,j}^{indirect}$$

where γ corresponds to a weight parameter (0 ≤ *γ* ≤ 1), which weighs the importance of direct trust relative to indirect trust feedback. This weight value then needs to be adjusted dynamically in order to effectively cope with various malicious attacks that may arise.

## 5. TASS-Based Service Composition in SIoT

The TASS score, which quantitatively integrates social strength and trust, enables the evaluation and ranking of candidate services for composite service planning in SIoT environments. This process can be implemented in both centralized and distributed architectures. In a centralized setting, a central server is responsible for aggregating interaction data and computing the relevant scores, which are then distributed to all participating nodes. In contrast, a distributed implementation allows each smart object to independently maintain its own trust estimates and periodically exchange information with neighboring devices, thereby supporting scalable and resilient trust management without a single point of failure. The choice between centralized and distributed deployment should be determined according to the specific requirements and scale of the intended IoT application.

γ corresponds to a weight parameter (0 ≤ *γ* ≤ 1), which weighs the importance of direct trust relative to indirect trust feedback. This weight value then needs to be adjusted dynamically in order to effectively cope with various malicious attacks that may arise.

Up to this point, we developed two independent measures (social strength and trust) that utilize the information available in SIoT networks. Social strengths between smart objects measure how “*co-workable*” two objects are at completing a task for users in general. On the other hand, trust signifies the expectation that interactions among objects will be satisfactory. We aim to support SIoT service composition applications by combining these two measurements to take part in the role of evaluating the performance of candidate composite services. The following illustrates the computation of the TASS score between Object *i* and Object *j*.

(12) $${TASS}_{i,j}=\lambda \cdot T_{i,j}+(1-\lambda )\cdot {ss}_{ij}$$

SIoT applications running on top of our TASS protocol must first create an SIoT service composition plan or a workflow of the desired composite service. A workflow of a composite service consists of atomic services connected by three basic types of workflow structures, namely sequential, parallel (AND), and selection (OR) [36]. Here, each atomic service has a list of candidate services from different service providers. Therefore, the applications are required to select the most trustworthy IoT nodes that provide the desired services such that the trust and social strength scores are maximized. Specifically, the combined TASS score of a composite SIoT service, denoted by ${score}_{i,j}^{TASS}$, that consists of two subservices (*j*1 and *j*2) depend on the structure connecting the two subservices, as follows:

- Sequential: ${score}_{i,j}^{TASS}={TASS}_{i,z}\times {TASS}_{z,j}$;
- Selection: ${score}_{i,j}^{TASS}=\max({TASS}_{i,j1},{TASS}_{i,j2})$;
- Parallel: ${score}_{i,j}^{TASS}=1-(1-{TASS}_{i,j1})\times (1-{TASS}_{i,j2})$.

In the subsequent section, we empirically validate the proposed service composition approach by applying the TASS protocol to real-world data, measuring utility and robustness across various configurations.

## 6. Experiments

### 6.1. Experimental Setting

To evaluate the performance and practical applicability of the proposed trust-augmented social strength (TASS) management protocol, we employed a real-world dataset collected from the Center for Advanced Studies in Adaptive Systems (CASAS) project at Washington State University. The CASAS project provides a rich repository of sensor-driven data collected from smart home environments, designed to support research in ambient intelligence, activity recognition, and behavior modeling.

The specific datasets used in our study contain detailed logs of daily activities performed by multiple participants residing in sensor-equipped smart homes. These activities include interactions with smart objects, such as using appliances, accessing cabinets, or turning lights on and off. Each object usage event is systematically annotated with the following information:

- A timestamp indicating the exact time of the interaction;
- A unique participant identifier (ID) to distinguish between different users;
- A unique object identifier (ID) representing the device involved in the interaction;
- A brief task description outlining the user’s intention or the context of the object usage (e.g., “preparing a meal”, “watching television”, etc.).

This rich contextual metadata enables us to extract human–object interaction sequences, which serve as the foundational input for constructing both object usage vectors and co-usage vectors. These vectors are further used in the computation of social strength values between smart objects, as outlined in the TASS framework.

The CASAS dataset is particularly well suited for this analysis due to its

- Realistic and naturalistic environment, where participants carry out their daily routines without artificial constraints;
- Temporal and behavioral diversity, offering a wide range of object usage patterns across different individuals and time spans;
- Well-structured format that facilitates temporal, spatial, and behavioral modeling required by the TASS protocol.

Moreover, the datasets provide the blueprints of the smart homes explicitly showing the locations of the objects. In total, there are 24 individual datasets with more than 600 participants who lived in the smart homes while they were monitored for their object usages. Out of all 24 datasets, we selected 4 of them to be our test datasets and the rest were used to generate the ground truth data. Here, we first grouped the objects in the smart homes based on the tasks that they were used for. For each task, we form friendship relations among the objects that belong to the task in accordance with our definition of social strength.

In order to analyze and compare the performance of the proposed approach with existing works, we used ns-3 (http://www.nsnam.org/ accessed on August 2022) as the simulator. We also allowed smart objects to behave maliciously as time progresses. The number of hostile objects, denoted by *MN*, were adjusted from 10% to 40% throughout the experiments. Note that there always exists noise in the IoT environment and, hence, the determination of the weight parameters is of utmost priority.

From a system architecture perspective, the actual evaluation of the scores at runtime is performed differently. In a centralized setting, the job is performed by the centralized server, whereas each distributed server ensures the evaluation of the scores in a distributed environment. By providing this option for system designers, they are granted flexibility in making design decisions.

The primary parameters (e.g., α, β, γ) in this study were determined based on prior literature and preliminary experiments. The impact of these parameters on system performance is briefly analyzed in Section 6.2, and a more comprehensive sensitivity analysis will be conducted in future work.

### 6.2. Results

#### 6.2.1. OPTIMAL α

In the first experiment, we investigated the influence of the parameter α in Equation (3) on the performance of our model, with the objective of identifying its optimal value. In this phase, Renyi diversity alone was used to compute the social strength between pairs of objects, allowing us to isolate the effect of α\alphaα on model behavior.

To conduct the experiment, we varied α across a continuous range from 0 to 1.5, and for each value, computed the normalized Renyi diversity for all object pairs in each of the four datasets. Since our ground truth labels only indicate the existence or absence of a social relationship (i.e., whether two objects are “friends”), we introduced a binary decision threshold, denoted by *θ*. Under this formulation, if the computed social strength between two objects exceeds *θ*, we infer the presence of a social link. For the purposes of this experiment, we fixed *θ* = 0.6; however, the optimal value of *θ* is explored in a subsequent experiment [34].

**Figure 1:**
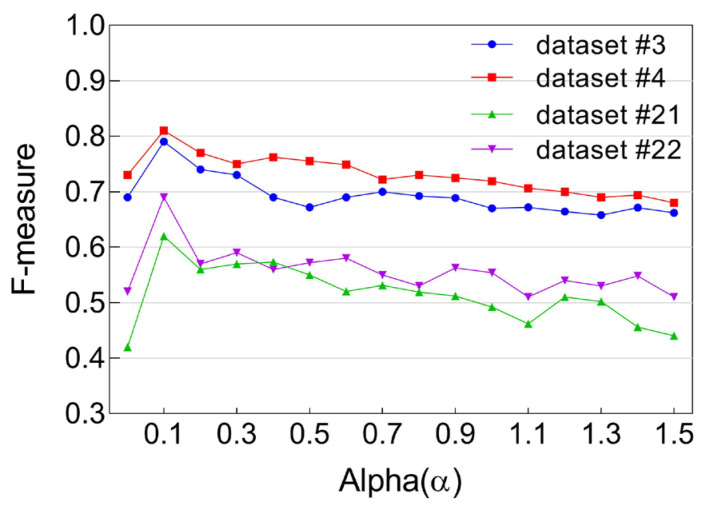
Impact of varying *α* on F-measure.

The predicted social ties—derived by comparing social strength values against the threshold—were then evaluated against the ground truth using the F-measure metric, which balances precision and recall. Figure 1 presents the resulting F-measure scores across varying values of α\alphaα.

From this analysis, we make the following key observations:

- **Peak Performance at α = 0.1**: All F-measure curves across the four datasets consistently peaked at α = 0.1, indicating that this value provides the most effective balance in moderating the influence of individual user co-usage frequencies on diversity. As such, α = 0.1 is identified as the optimal setting for controlling skewness caused by extreme co-usage behaviors.
- **Degenerate Case at α = 0**: When α is set to zero, the Renyi diversity measure reduces to a simple count of unique users, effectively ignoring repeated co-usage events by the same user. This oversimplification leads to underrepresentation of important behavioral patterns, and consequently, lower F-measure scores.
- **Diminishing Returns for High α Values**: As α increases beyond 0.1 toward 1.5, we observe a gradual decline in model performance. This decline stems from the increasing sensitivity of Renyi diversity to **high-frequency outliers**—instances where a small number of users exhibit disproportionately high co-usage. These outliers begin to dominate the diversity score, skewing social strength calculations and resulting in a rise in false positives.
- **Over-Limiting at Very Low α Values**: Conversely, decreasing α below 0.1 leads to over-suppression of frequency influence. While this helps reduce the effect of outliers, it also **dampens the contribution of moderately frequent co-usage events**, which are essential for robust diversity estimation. This over-limiting effect degrades model accuracy and illustrates the importance of selecting a balanced α.

Based on these findings, we fixed the parameter α\alphaα to **0.1** for all subsequent experiments, as it consistently delivered the **highest F-measure scores** across all datasets.

#### 6.2.2. OPTIMAL Threshold θ and β

The second experiment focuses on determining the optimal threshold value *θ*, which is used to decide whether a computed social strength score between two smart objects signifies a valid friendship relationship. While Equation (6) is proposed as the final formula for computing social strength, its effectiveness depends on selecting appropriate weighting parameters *β* and 1 − *β*, which balance the contributions of Renyi diversity and MND-based frequency, respectively.

To estimate the optimal values of these parameters, we leveraged structural similarity metrics widely used in graph-based link prediction: Jaccard’s Index, Adamic/Adar similarity, and the Katz score. These metrics were applied over a social graph constructed from the ground truth data, where nodes represent smart objects and edges represent known friendship ties. Each metric computes a reference value of social strength for every object pair, which we then used as a target variable in a linear regression model with Renyi diversity and MND-based frequency as independent variables [37].

Using the least squares method, we derived the optimal weight coefficients (*β*, 1 − *β*) for each similarity metric:

- Jaccard Index: *β* = 0.424, 1 − *β* = 0.576;
- Adamic/Adar Similarity: *β* = 0.461, 1 − *β* = 0.539;
- Katz Score: *β* = 0.478, 1 − *β* = 0.522.

These results reveal an important insight: in all three cases, the **weight assigned to MND-based frequency (1 − *β*)** exceeds that of Renyi diversity. This trend suggests that emphasizing spatial proximity plays a critical role in **mitigating the effects of data sparsity**, particularly when usage patterns are unevenly distributed.

**Figure 2:**
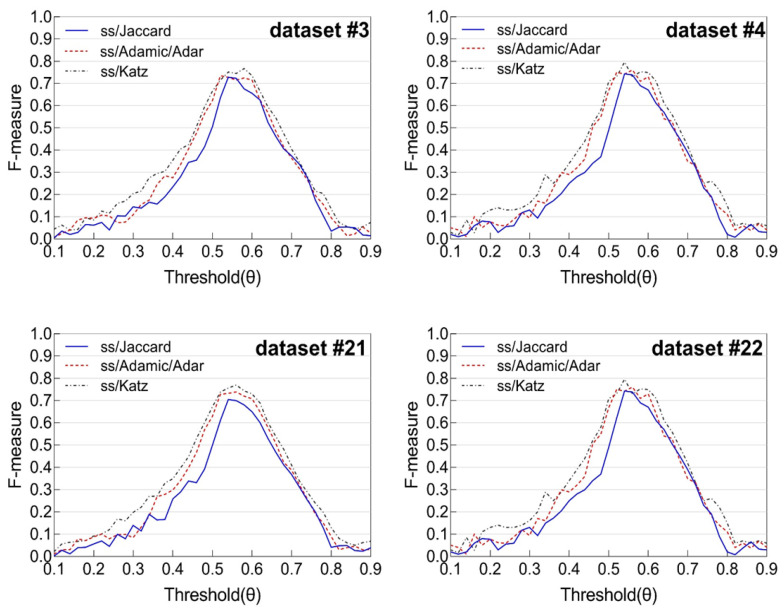
F-measure of Jaccard, Adamic/Adar, and Katz scores in terms of varying threshold *θ*.

After incorporating these learned coefficients into Equation (6), we computed the final social strength scores for all object pairs across the four datasets. To evaluate the performance of each weighting scheme, we varied the threshold *θ* and plotted the corresponding **F-measure scores**. The results of this experiment are illustrated in Figure 2.

Among the three weighting strategies, the configuration derived from the **Katz score** consistently produced the **highest F-measure values** across all datasets. Furthermore, we observed that the F-measure curves for all datasets reached their peak when the threshold *θ* was set to approximately **0.54**. This threshold appears to offer the best trade-off between precision and recall in identifying socially linked object pairs.

In summary, the experimental results demonstrate that the **Katz-based weighting scheme**, combined with a decision threshold of *θ* = 0.54, yields the most accurate performance in detecting social ties. Accordingly, we adopt these values for the remaining experiments throughout the study.

#### 6.2.3. Precision vs. Recall

**Figure 3:**
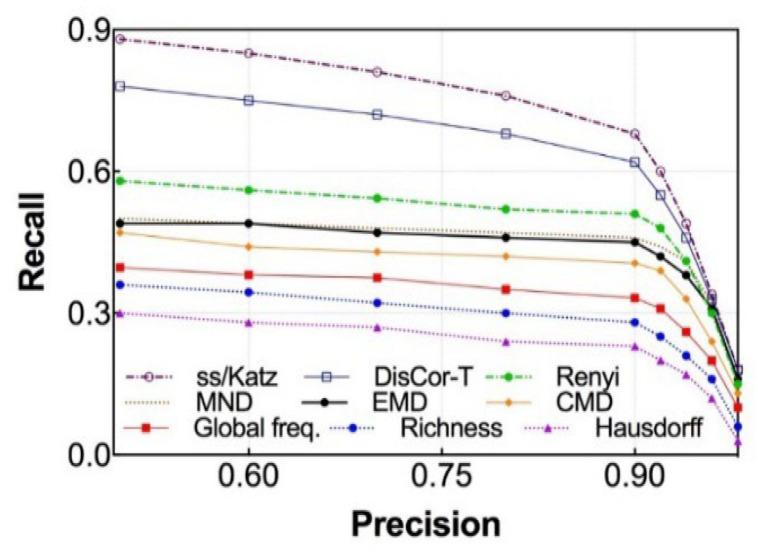
Precision vs. recall comparison with other methods.

Based on the findings from the previous experiments, we compare the precision and recall of our social strength model with some of the related approaches as shown in Figure 3. As one might predict, we used the optimal values of parameters, *α* = 0.1, (*β*, *γ*) = (0.478, 0.522), and *θ* = 0.54 for this particular experiment. Note that we do not cover what each of the methods introduced in Figure 3 is in this work, but the detailed descriptions of these approaches can be found in [38].

Overall, the proposed **social strength computation framework**, when integrated with the **Katz score-based weighting scheme**, demonstrated superior performance compared to all other baseline methods evaluated in this study. These baselines included traditional graph-based similarity metrics that do not explicitly incorporate temporal or spatial dimensions of object interactions.

Quantitatively, the model achieved a **precision of 0.83** and a **recall of 0.72**, resulting in an **F-measure of 0.77**—a balanced indicator that reflects both the accuracy and completeness of social tie detection. This level of performance indicates that the proposed approach is not only capable of identifying a high proportion of true positive relationships (i.e., precision), but also effective at discovering the majority of relevant ties present in the ground truth data (i.e., recall).

The performance gains can be attributed to the **multi-dimensional nature** of our framework. Unlike conventional similarity-based techniques, our approach integrates

- **User diversity**, captured through Rényi entropy, which reflects the generalizability of co-usage patterns across different individuals;
- **Spatial proximity**, quantified via the Mutually Nearest Distance (MND), which captures the likelihood of physical co-occurrence of object usage;
- **Temporal alignment**, incorporated through co-usage vector construction within specific time intervals, which infers task-level coordination between objects.

By jointly leveraging these behavioral and contextual features, the model effectively captures **latent social relationships** that may be overlooked by methods relying solely on topological or frequency-based cues. This holistic view of object interaction dynamics is what enables our framework to outperform alternative strategies in terms of both **precision and recall**, thus establishing its practical value for social relationship inference in SIoT systems.

#### 6.2.4. Trust Convergence, Accuracy, and Resiliency

In this section, we examine the trust convergence, accuracy, and resiliency properties of our TASS management protocol design. We first perform a comparative analysis on static control (i.e., *γ* is fixed at a constant) versus adaptive control (i.e., *γ* is changed dynamically based on (11)) when *MN* is kept at 20% throughout the course of the evaluation.

**Figure 4:**
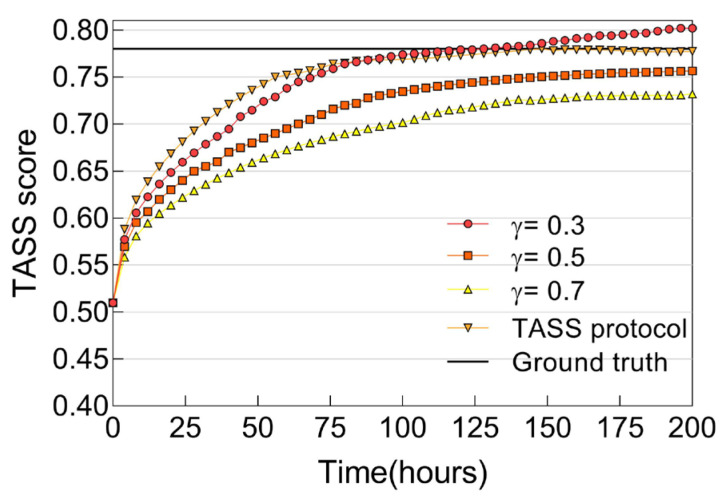
TASS score of a good node with respect to varying *γ*.

Figure 4 shows TASS score evaluation results for a trustor node toward a non-malicious trustee node that is randomly selected. Here, we can observe that trust convergence is achieved for both fixed and adaptive control. There is a trade-off between convergence time and trust bias. For a given trustor node, the former measures how quickly it reaches the ground truth TASS score, whereas the latter measures how close its TASS score is to the ground truth after convergence. In cases of static control, the trust convergence time becomes longer and the trust bias becomes smaller when a larger *γ* value is used. For adaptive control, on the other hand, the trustor node is able to adjust *γ* dynamically in order to minimize both the convergence time and the trust bias after convergence. In Figure 4, an interesting observation to note is the case where *γ* is too small (i.e., 0.3). In this particular case, the TASS score is over-estimated upon convergence, which certainly is not a desirable trait as trust overshooting is considered detrimental to the stability of any trust management system. Our TASS protocol, however, adjusts the value of *γ* dynamically for fast convergence without incurring the trust overshooting phenomenon.

**Figure 5:**
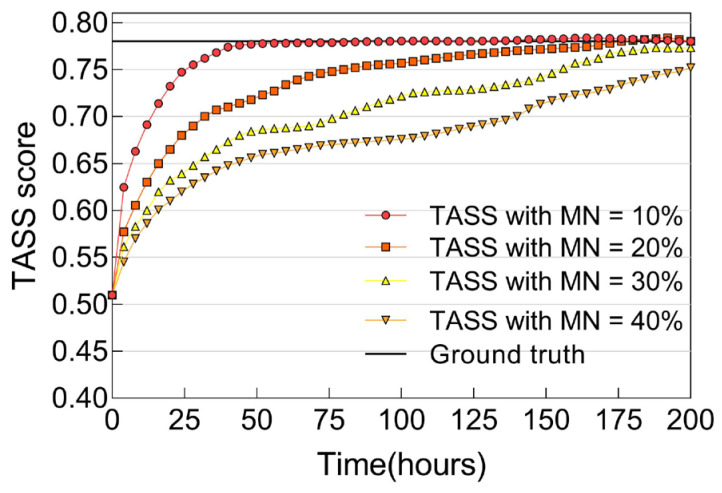
TASS score of a good node with respect to varying *MN*.

The next experiment tests the resiliency of the proposed TASS protocol against a malicious node population. In particular, we evaluate how the TASS score for a trustor node toward a well-behaved trustee node change with respect to varying percentages of *MN*. In Figure 5, we can observe that as the population of malicious nodes increases, both the convergence time and trust bias increase. However, the TASS protocol is found to be resilient to various malicious attacks for *MN* up to 40 percent, with the graph illustrating proper trust convergence and accuracy behaviors. Overall, we observe that the trust bias is kept at a minimum when *MN* ≤ 40%. Therefore, this depicts the resiliency property of the proposed TASS protocol against malicious attacks in SIoT.

#### 6.2.5. Utility Score of Service Composition

In order to verify whether the proposed TASS protocol, in fact, enhances the performance of service composition, we test two different cases where service composition is carried out without/with QoS constraints.

**Figure 6:**
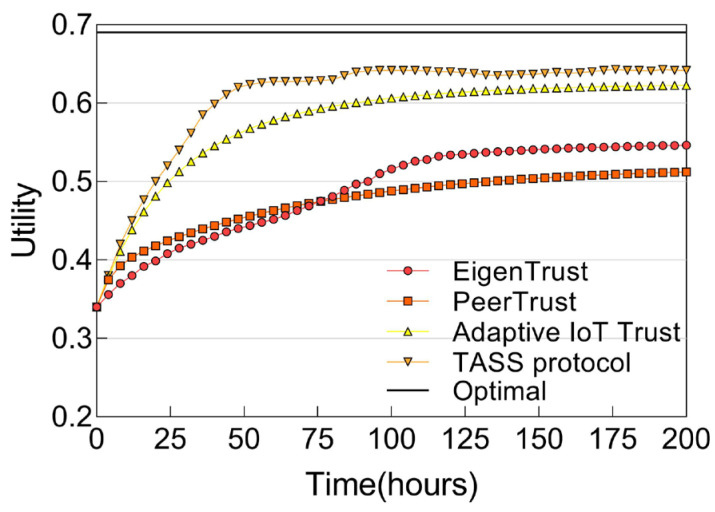
Utility score of IoT service composition without constraints.

Figure 6 shows the ns-3 simulation results showing the utility scores of IoT service composition with *MN* = 20%. We observe that the trust-based service composition with our TASS protocol significantly outperforms random service composition and upon convergence approaches the performance of ideal service composition based on ground truth. Furthermore, our TASS protocol outperforms EigenTrust [25], PeerTrust [26], and adaptive IoT trust [19] as the underlying trust protocol for trust-based service composition. Finally, we also observe that the performance gap widens as the percentage of *MN* increases.

For the case where we impose QoS constraints, simply selecting the most trustworthy service provider (i.e., smart object) may lead to infeasible solutions [7]. In trust-based service composition with constraints, we calculate the overall utility score and the overall QoS constraint for each candidate configuration, and select the configuration with the highest utility score with the overall QoS constraint conforming to the global QoS constraint.

**Figure 7:**
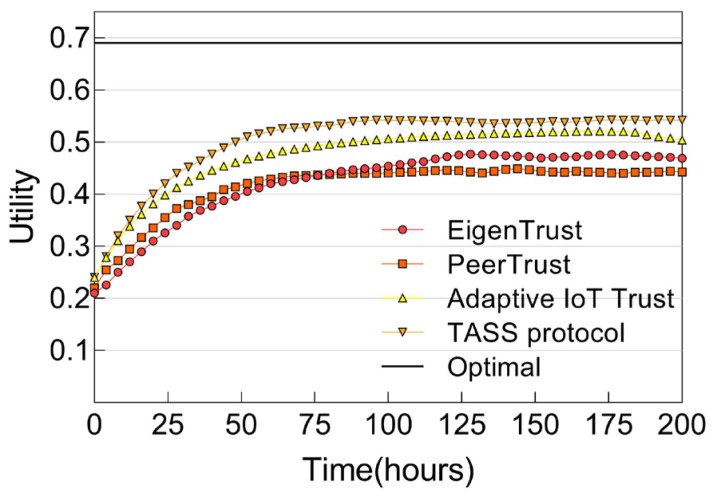
Utility score of IoT service composition with constraints.

In Figure 7, we demonstrate the ns-3 simulation results when constraints are placed with *MN* = 20%. We first observe that the utility scores are lower than those without QoS constraints since good service providers may post strict QoS constraints, thereby preventing them from being included. Here, we observe that the trend is similar to Figure 6 in terms of performance ranking, with our TASS protocol outperforming all of the conventional techniques. We attribute the superiority of our protocol over these methods to our protocol’s adaptability in response to a high percentage of nodes performing malicious attacks and the utilization of social strength values among smart objects.

Despite the advantages of the proposed approach, our work still has some room for improvement. Most importantly, we believe that social strengths among smart objects should be represented more naturally with respect to time. In other words, the evolution of social strengths should preferably be captured more dynamically by incorporating a decaying factor [39]. By doing so, we believe that fluctuations in how closely related (i.e., social strength) two objects are can be precisely modeled not just at a given moment in time but over a time interval.

Furthermore, we recognize that there may be additional factors influencing the correlation between the social strengths of two objects in an IoT environment. For instance, many smart objects, such as smartphones, are often owned by individuals, and in human-centric social networks, these individuals are connected through friendships. Generally, friends tend to share similar interests and daily routines, suggesting a potential correlation between the social strength of two objects and the closeness of the people who use them as friends [40,41,42]. Of course, this assumption requires further validation through enhancements to our social strength computation algorithm and more rigorous experiments.

The experimental results show that the proposed TASS protocol achieves improved trust estimation accuracy and robustness compared to existing baseline methods under various simulated attack scenarios. However, it should be noted that the current validation is limited to a specific dataset and simulated environment.

## 7. Conclusions and Future Work

In this study, we thoroughly examined the challenge of quantifying social strength and trust among smart objects in order to support resilient and accurate service composition within Social Internet of Things (SIoT) environments. To this end, we introduced the TASS management protocol, which utilizes co-usage data to assess social strength, while simultaneously integrating both the experience of individual objects and peer recommendations to enable dynamic trust estimation. This dual approach was designed to effectively address common issues in SIoT, such as the sparsity and imbalance of usage data, as well as the need for adaptability and robustness against various threats.

Our experimental evaluation demonstrated that TASS achieves superior performance compared to state-of-the-art techniques, showing improvements in precision, recall, trust convergence rate, resiliency under simulated attack scenarios, and the utility scores achieved in IoT service composition tasks. These results suggest that TASS provides a robust and scalable framework for managing trust and social strength in complex SIoT environments.

However, while the findings are promising, our work is not without limitations. Most notably, the current validation is confined to the CASAS smart home dataset, which may not fully reflect the diversity and scale of real-world SIoT systems. Moreover, although TASS exhibited enhanced robustness under simulated attack conditions, the computational overhead and real-time scalability of the protocol in very large or highly heterogeneous networks were not extensively evaluated within this study.

Looking ahead, we recognize the need for more comprehensive validation and broader experimentation. Future research will focus on applying TASS to additional public SIoT datasets, as well as deploying it in various application domains to assess its generalizability and effectiveness. We also plan to enhance the protocol’s suitability for large-scale distributed environments, and to integrate it with other service computing functions such as service discovery and recommendation. Additionally, we aim to further investigate ways to mitigate trust fluctuations and maximize utility in service composition. There is also significant potential in incorporating the dynamic modeling of social relationships and leveraging advanced graph prompt learning techniques to further enrich the protocol’s capabilities [43,44,45]. Most importantly, we intend to test the practical applicability and scalability of TASS in real-world IoT environments, utilizing larger and more diverse datasets from multiple domains.

Through these future efforts, we hope to build upon the foundation established in this work and further advance the reliability and intelligence of SIoT service composition.

## Data Availability

Data sharing is not applicable to this article.
